# Effects of anastrozole on Ki-67 antigen expression in the vaginal epithelium of female rats in persistent estrus

**DOI:** 10.6061/clinics/2020/e1643

**Published:** 2020-03-26

**Authors:** Diego Cipriano Chagas, Maria da Conceição Barros-Oliveira, Pedro Vitor Lopes-Costa, Renato de Oliveira Pereira, Mariella de Almeida Melo, Danylo Rafhael Costa-Silva, Carine Soares Borges, Jackeline Lopes Viana, Alesse Ribeiro dos Santos, Gil Facina, Benedito Borges da Silva

**Affiliations:** I Programa de Pos Graduacao em Ciencias da Saude, Universidade Federal do Piaui, Teresina, PI, BR; II Programa de Pos Graduacao, Rede Nordeste de Biotecnologia (RENORBIO), Universidade Federal do Piaui, Teresina, PI, BR; III Hospital Getulio Vargas, Universidade Federal do Piaui, Teresina, PI, BR; IV Departamento Ginecologia, Universidade Federal de Sao Paulo, Sao Paulo, SP, BR

**Keywords:** Anastrozole, Rat, Vagina, Proliferation, Ki-67

## Abstract

**OBJECTIVES:**

Aromatase inhibitors are the first-choice drugs for the treatment of hormone sensitive breast cancer. However, in addition to the scarcity of studies, there are controversies about their effects on vaginal epithelial cell proliferation in rats, especially those in persistent estrus.

**METHODS:**

To investigate vaginal epithelial cell proliferation by Ki-67 antigen expression, persistent estrus was induced in 42 randomly selected rats. These rats were randomly divided into 2 groups: group I (control, n=21), which received 0.1 mL of propylene glycol (vehicle) daily, and group II (experimental, n=21), which received 0.5 mg/kg or 0.125 mg/day of anastrozole diluted with 0.1 mL of propylene glycol.

**RESULTS:**

Light microscopy showed a higher concentration of cells with brown Ki-67 stained nuclei in the control compared to the experimental group. The mean percentage of Ki-67 stained nuclei per 500 cells in the vaginal epithelium was 68.64±2.64 and 30.46±2.00 [mean±standard error of the mean (SEM)] in the control and experimental groups, respectively (*p*<0.003).

**CONCLUSION:**

This study showed that anastrozole, at the dose and treatment duration selected, significantly decreased cell proliferation in the vaginal mucosa of the rats in persistent estrus.

## INTRODUCTION

Vaginal atrophy and hormone-sensitive breast cancer are common conditions in postmenopausal women, for which tamoxifen is the standard endocrine therapy; however, although tamoxifen may have trophic effects on the vaginal epithelium, severe adverse effects such as endometrial cancer lead to the search of alternative endocrine therapies 1. Third-generation aromatase inhibitors, such as anastrozole, letrozole, and exemestane, have become the first-choice drug for endocrine treatment of hormone-sensitive breast cancer in postmenopausal women. In particular, anastrozole has been emphasized as the endocrine therapy of choice in postmenopausal women 2. These drugs are associated with greater efficacy in breast cancer and improved general tolerability compared to tamoxifen 3. Furthermore, tamoxifen is associated with undesirable adverse effects, particularly, the increased incidence of endometrial hyperplasia and even endometrial cancer, as well as cataracts, thromboembolism, and cerebrovascular events 4.

The use of anastrozole for long periods does not show any effect on the levels of steroid hormones, such as cortisol, aldosterone, androstenedione, and 16-hydroxyprogesterone, confirming anastrozole's high selectivity in inhibiting aromatase without interfering with other adrenal steroidogenesis pathways 5-7. Anastrozole also does not affect the synthesis of gonadotropins and steroids that are dependent on the hypothalamic-pituitary-ovarian axis 8-10. Studies on the effects of anastrozole on the vaginal epithelium in women present ethical limitations. Hence the need for experimental animal models exists, even though there are limitations in regards to the extrapolation of results from animals to humans 11-13. In terms of vaginal epithelial tissue and sensitivity to therapy, the rat animal model would be most similar to humans 14.

The persistent estrus female rat is a model that is under constant estrogenic stimulation, mimicking polycystic ovarian syndrome. Therefore this model is interesting and useful to study the effects of hormonal drugs 15-16, such as anastrozole, which inhibit estrogen synthesis. Nery-Aguiar et al. 1 showed that tamoxifen significantly increased cell proliferation in the vaginal mucosa of castrated rats, as evaluated by means of Ki-67 protein expression. On the other hand, the effects of anastrozole in the vaginal epithelium of premenopausal rats are unclear. Meanwhile, among the aromatase inhibitors, letrozole significantly reduced cell proliferation in the endometrial and vaginal epithelia of female rats 17. However, Sadlonova et al. 18 administered anastrozole in the diet of rats for 15 weeks and did not show atrophy of the endometrial and vaginal epithelia. Although these were experimental studies using animal models, they lead to further interest for studies aimed at the clinical application of aromatase inhibitors in women with breast cancer and vaginal atrophy. Considering the controversies and, to the best of our knowledge, the scarcity of studies of the effects of anastrozole on the vaginal epithelium of rats in persistent estrus, the present study was designed.

## MATERIALS AND METHODS

### Animals

This study was approved by the Animal Experimentation Ethics Committee of the Federal University of Piauí (UFPI) and conducted according to the ethical principles established by the Brazilian College of Animal Experimentation (COBEA). We used 42 Wistar-Hannover rats, weighing approximately 250g each, from the Veterinary Sciences Laboratory of the Federal University of Piauí. Persistent estrus was induced in the animals through a subcutaneous injection of 1.25 mg of testosterone propionate on the second day of life. During the study, all animals were housed in plastic cages with metal tops (grills) at an ambient temperature ranging from 20°C to 24°C. Fluorescent lamps provided 12-h light/12-h dark cycles. While their food portions were rationed, the rats had free access to filtered water. At 90 days of life, androgenized rats were selected for research. Persistent estrus rats presented with occlusion of the distal third of the vagina and keratinization of the vaginal epithelium (the main characteristic of persistent estrus); the presence of polycystic ovaries was noted at the time of autopsy 15. The animals were randomly divided into two groups: group I (control, n=21) and group II (experimental, n=21). Each rat from Group I (control) received 0.1 mL/day of propylene glycol (vehicle) and each rat from experimental group II received 0.5 mg/kg or 0.125 mg/day of anastrozole diluted with 0.1 mL of propylene glycol 17. The vehicle and anastrozole were administered by oral gavage continuously for 28 days, at the same time of each day (between 15:00 and 16:00). On the 29^th^ day, the rats from both groups were euthanized by an intraperitoneal injection containing an excessive dose of anesthetics, 300 mg/kg ketamine and 15 mg/kg midazolam. The rats were then immobilized on a cork board to remove the vagina, and the tissue was fixed in buffered formalin (pH approximately 7.5). After 24h fixation, the vaginal epithelium was subjected to immunohistochemical analysis.

### Immunohistochemistry

Histological sectioning to produce the histological slides was performed using the Minota-type microtome adjusted to 5-μm thickness. The preparation of the histological slides and respective immunohistochemistry, were performed simultaneously under the same conditions. Immunohistochemical evaluation of the Ki-67 marker was performed using a detection system combined with an antigen retrieval method. For this, the sections were treated with 3% hydrogen peroxide diluted in buffered solution for 5 min to block the endogenous peroxide. Following antigen recovery, tissue samples were incubated with anti-Ki-67 rat primary monoclonal antibody (clone MIB-5/1:100) for 16h overnight in a refrigerator at approximately 4°C. The samples were then washed with buffered saline and incubated for 45 min with the New Link Polymer detection system. To read these reactions, all histological slides were treated for 5 min with a 3-3 solution of benzidine diamine tetrahydrochloride at a concentration of 1 mg/mL of Tris buffered saline and hydrogen peroxide solution, then contrasted with Harris Hematoxylin for 5 min, followed by dehydration in ethyl alcohol and xylol baths. The cells were considered positive for immunohistochemical expression of the Ki-67 antigen when their nuclei were stained with a brown color.

### Quantitative method

Cell counting was performed manually by two blind observers in relation to the groups studied, and the positive and negative cells were labeled separately to avoid counting the same cells more than once. Cell counting was performed at a research laboratory located at the Gynecological Coordination of the Getulio Vargas Hospital/Federal University of Piaui, where a computerized system consisting of a Light Microscope Eclipse E-400 (Tokyo, Japan) was used, coupled with a color camcorder (Samsung Digital Camera SCC-131, Seoul, South Korea). Images were captured on a Pentium IV microcomputer with an 80-Gigabyte hard drive, 3.0-GHz processor, 1024 RAM, and graphics card using Windows XP. Cell counting for Ki-67 positive and negative cells was performed at 400× magnification. At least 500 cells in the vaginal epithelium were counted in each slide in random fields, starting in the area of higher nuclei concentration with Ki-67 expression, using Imagelab^®^ Image Analysis and Processing Software (SOFTIUM Informática LTDA, São Paulo, Brazil).

### Statistical Analysis

The data were analyzed using the non-parametric Mann–Whitney test. The data were tabulated using the IBM SPSS Statistics V21 program, with the significance level set at *p*<0.05.

## RESULTS

**Figure 1:**
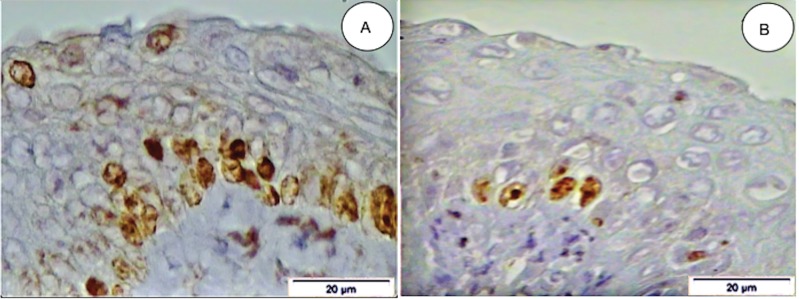
Photomicrography of a histological section of the vaginal epithelium from a female rat in persistent estrus. Note the presence of a higher concentration nuclei stained brown by anti-Ki-67 (MIB-5) antibody prior to treatment with anastrozole, control group (A), and sparsely stained nuclei post-treatment, experimental group (B) (original magnification=400x).

**Figure 2:**
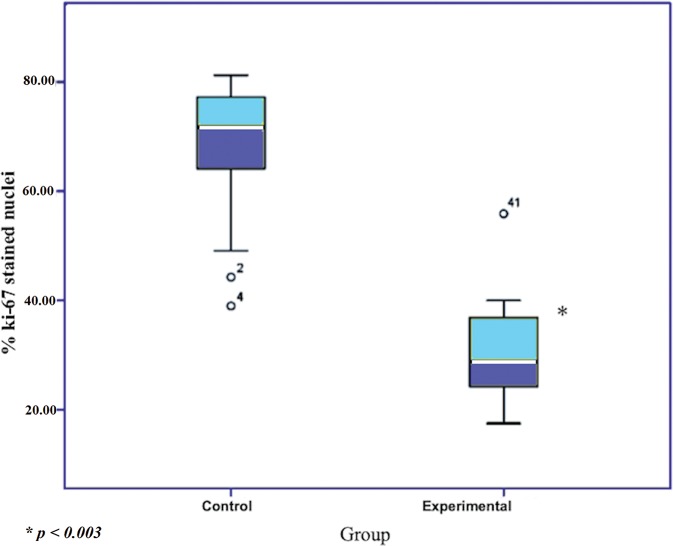
Box plot clearly showing the difference between the mean percentage of Ki-67 stained nuclei in the control and experimental groups.

Under light microscopy, the concentration of cells expressing the Ki-67 antigen was higher in the control group compared to the experimental group (Figure 1). The mean numbers (±standard error [SE] of the mean) of Ki-67-stained nuclei in the vaginal epithelium of rats in persistent estrus were 68.64±2.64 and 30.46±2.00 in groups I (control) and II (experimental), respectively (*p*<0.003) (Table 1). Figure 2 clearly shows the difference between the mean percentage of Ki-67-stained nuclei in the control and experimental groups.

## DISCUSSION

Studies on the effects of drugs used to treat hormone-sensitive breast cancer in human tissue are limited for ethical reasons; hence experiments using animal models are reasonable alternatives. This led us to study the effects of anastrozole, an inhibitor of the synthesis of estrogens, in a rat experimental model where the rats are in persistent estrus as the vaginal epithelium is under constant estrogen stimulation. In the present study, rats in persistent estrus (control) showed a higher concentration of Ki-67-stained nuclei in the epithelium compared to the group treated with anastrozole. The mean number of Ki-67-stained nuclei was significantly reduced in the group treated with anastrozole than in the control group.

The persistent estrus female rat is an animal model that mimics polycystic ovarian syndrome. The female rat in persistent estrus an intensely proliferated vaginal epithelium with vaginal cornification, a characteristic that defines this experimental model 15. The persistent estrus female rat animal model is an interesting model to study molecules that inhibit the synthesis of estrogens, such as aromatase inhibitors. Kubtka et al. 17 showed high tumor suppressive effects of letrozole in a premenopausal model of mammary carcinogenesis in female rats.

In this study, anastrozole was administered by oral gavage, which, although more laborious, is similar to the route commonly used by human females. In postmenopausal women, anastrozole is generally administered at a dose of 1 mg/day for breast cancer treatment 6,19. However, there are differences in drug absorption and metabolism between humans and animals, since rats have a more rapid metabolism 20. Considering these aspects, administration by oral gavage allowed the animals to receive a selected dose of drug more similar to that in humans 21. We administered an anastrozole dose of 0.5 mg/kg per day or 0.125 mg/animal/day based on studies by Kubatka et al. 22 and Sadlonova et al. 18, who observed that a dose of 0.5 mg/kg administered to rats is similar to a daily clinical dose of 1 mg in postmenopausal women with breast cancer.

Some studies have evaluated cell proliferation in the mammary, uterine, and vaginal epithelia of rats based on the expression of the Ki-67 antigen. Gerdes et al. 23 found a Ki-67 antibody that recognized a nuclear antigen present in proliferating cells and absent in resting cells, making it ideal for the evaluation of cell proliferative activity. Anastrozole is administered in postmenopausal women with hormone-sensitive breast cancer; however, it has been shown that aromatase inhibitors may lead to adverse effects in premenopausal female rats, such as dietary exemestane-induced mammary carcinogenesis 24. Nevertheless, the effects of anastrozole administered by oral gavage to premenopausal rats, as opposed to oral exposure in the diet, are unknown. Meanwhile, Nery-Aguiar et al. 1 administered tamoxifen to castrated female rats, mimicking postmenopausal women, which increased proliferation in the vaginal epithelium. On the other hand, Sousa-Lages et al. 25 did not show alterations in the vaginal epithelium of castrated female rats with the use of pilocarpine, a cholinergic parasympathomimetic agonist that induces an increase in glandular secretion and with controversial effects on vaginal dryness.

The few studies that do exist on the effects of aromatase inhibitors in the uterine and vaginal epithelia of rats present controversial results. Kubatka et al. 17 analyzed the preventative effects of letrozole in rats and found that letrozole leads to uterine and vaginal atrophy, increased concentrations of plasma triacylglycerols, and increased body weight. In contrast to these results, in two other studies, Kubatka et al. 22 analyzed the chemopreventive effects of anastrozole and showed that the drug suppressed breast tumor incidence by 40%; however, its effects were not shown on the genital system (uterus and vagina) of these rats. The same authors analyzed the effects of exemestane on the histopathology of the uterus and vagina; the study also did not reveal alterations, which points to the non-antiestrogenic effects of the drug 24. Sadlonova et al. 18 also analyzed the effects of anastrozole; their histological examination did not show atrophic changes in the endometrium of the uterus and vaginal epithelia.

According to the aforementioned studies, the drugs were administered in the animal feed, and as reported, letrozole caused uterine and vaginal atrophy. These authors also did not identify any vaginal alterations after administering exemestane and anastrozole in the animal feed. On the other hand, Barros-Oliveira et al. showed significantly decreased expression of cell proliferation based on Ki-67 expression in the mammary epithelium of rats in persistent estrus receiving 0.125 mg daily of anastrozole by oral gavage for 28 days 20. Furthermore, Ferreira et al. 26 and Mahamed et al. 27 showed a reduction in uterine cell apoptosis and endometrial thickness of rats in persistent estrus treated with melatonin and metformin, respectively.

Thus, we conclude that anastrozole administered to female rats in persistent estrus at a dose of 0.5 mg/kg/day, that is 0.125 mg/day, by oral gavage for 28 days, significantly decreased Ki-67 antigen expression in the vaginal epithelium, showing an anti-proliferative effect of anastrozole on the vaginal mucosa. However, further studies are necessary and important to confirm these scientific findings.

## AUTHOR CONTRIBUTIONS

Chagas DC, Barros-Oliveira MC, Lopes-Costa PV and da Silva BB conceived and designed the study, and wrote the final version of the manuscript. Pereira RO, Melo MA, dos Santos AR, Costa-Silva DR, Borges CS, Viana JL, Facina G, da Silva BB were responsible for conducting the study, statistically analyzing the data, and manuscript drafting and review. All of the authors reviewed the manuscript.

## Figures and Tables

**Table 1:** Mean percentage of Ki-67 nuclei per 500 cells in the control an experimental groups.

		% Ki-67-stained nuclei				
		Mean	SE	Median	Minimum	Maximum
Group	Control	68.64	2.64	71.76	38.98	81.21
	Experimental	30.46*	2.00	28.71	17.47	55.85

* There was a statistically significant decrease in Ki-67-stained nuclei after treatment with aanastrozole (*p*<0.003).
